# Supplementary material for: Interaction Between Human Skeletal and Mesenchymal Stem Cells Under Physioxia Enhances Cartilage Organoid Formation: A Phenotypic, Molecular, and Functional Characterization
Source: Cells. 2025 Sep 11;14(18):1423. doi: 10.3390/cells14181423 (PMC12468709; doi:10.3390/cells14181423)
Supplement: Supplementary file 1 [file cells-14-01423-s001.zip › cells-3844226-supplementary.pdf]

## Supplements

**Table S1.** Primers for Pluripotency, Multipotent Capacity, and Chondrogenic Differentiation

| Assay | Gene | Primers | Size |
|---|---|---|---|
| **Pluripotency** | **NANOG** | FW: 5' AGTCCCAAAGGCAAACAACCCACTTC 3'<br>RV: 5' TGCTGGAGGCTGAGGTATTTCTGTCTC 3' | 161 pb |
| | **OCT3/4** | FW: 5' GGGGTTCTATTTGGGAAGGTAT 3'<br>RV: 5' GTTCGCTTTCTCTTTCGGGC 3' | 191 pb |
| | **TRA1-81** | FW: 5' GTAACTGGGCAAAGTGTG 3'<br>FW: 5' GTAACTGGGCAAAGTGTG 3' | 91 pb |
| **Multipotent Capacity** | **RUNX2** | FW: 5′ GGAATGCCTCTGCTGTTATG 3′<br>RV: 5′ AGGATTTGTGAAGACGGTTATG 3′ | 116 pb |
| | **PPARɣ** | FW: 5' TCTCTCCGTAATGGAAGACC 3'<br>RV: 5' GCATTATGAGACATCCCCAC 3' | 473 pb |
| | **SOX9** | FW: 5' AGGAAGTCGGTGAAGAACGG 3'<br>RV: 5' AAGTCGATAGGGGGCTGTCT 3′ | 274 pb |
| **Chondrogenic Differentiation** | **COL2A1** | FW: 5' CAACAACCAGATTGAGAGCA 3'<br>RV: 5' CCATGTTGCAGAAAACCTTC 3' | 168 pb |
| | **ACAN** | FW: 5′ GGAGCAGGAGTTTGTCAACA 3′<br>RV: 5′ CTTCTCGTGCCAGATCATCA 3′ | 187 pb |
| | **SOX9** | FW: 5' AGGAAGTCGGTGAAGAACGG 3'<br>RV: 5' AAGTCGATAGGGGGCTGTCT 3′ | 274 pb |
| | **PGR4** | FW: 5′ GTAGATGAAGCTGGAAGTGG 3′<br>RV: 5′ CTGGGTCTGGGATTTATTGG 3′ | 134 pb |
| | **PDPN** | FW: 5′ GAAAGTGGATGGAGACACACA 3′<br>RV: 5′ CAATGAAGCCGATGGCTAGTA 3' | 104 pb |
| | **RUNX2** | FW: 5′ GGAATGCCTCTGCTGTTATG 3′<br>RV: 5′ AGGATTTGTGAAGACGGTTATG 3′ | 116 pb |
| | **COL10** | FW: 5′ GCTAAGGGTGAAAGGGGTTC 3′<br>RV: 5′ CTCCAGGATCACCTTTTGGA 3′ | 118 pb |
| **Housekeeping genes** | **GAPDH** | FW: 5' ACAACTTTGGTATCGTGGAAGG 3'<br>RV: 5' GCCATCACGCCACAGTTTC 3' | 101 pb |
| | **B2M** | FW: 5' GCTGTCTCCATGTTTGATGTATC 3'<br>RV: 5' TCTCTGCTCCCCACCTCTAAGT 3' | 89 pb |
| | **ACTB** | FW: 5' GCCCTGGCACCCAGCACAAT 3'<br>RV: 5' AGGGGCCGGACTCGTCAT 3' | 148 pb |

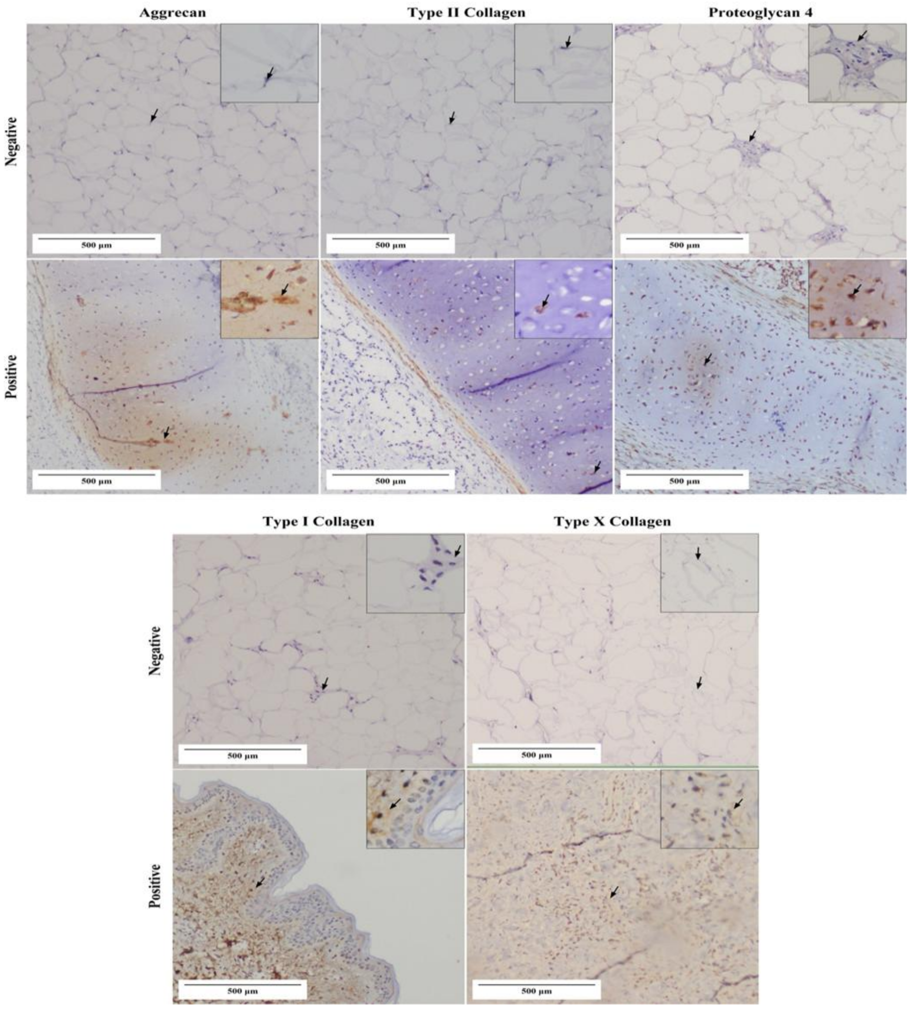


**Figure S1.** Extracellular Matrix (ECM) Proteins in Control Tissues. (A) Negative expression of aggrecan, type II collagen, and proteoglycan-4 in adipose tissue; positive expression of aggrecan, type II collagen, and proteoglycan-4 in fetal tracheal cartilage. (B) Negative expression of type I and type X collagen in adipose tissue; positive expression of type I collagen in the skin and type X collagen in the intervertebral disc. Images acquired using OLYMPUS DP22 microscope, 10X objective; inset at 40X objective

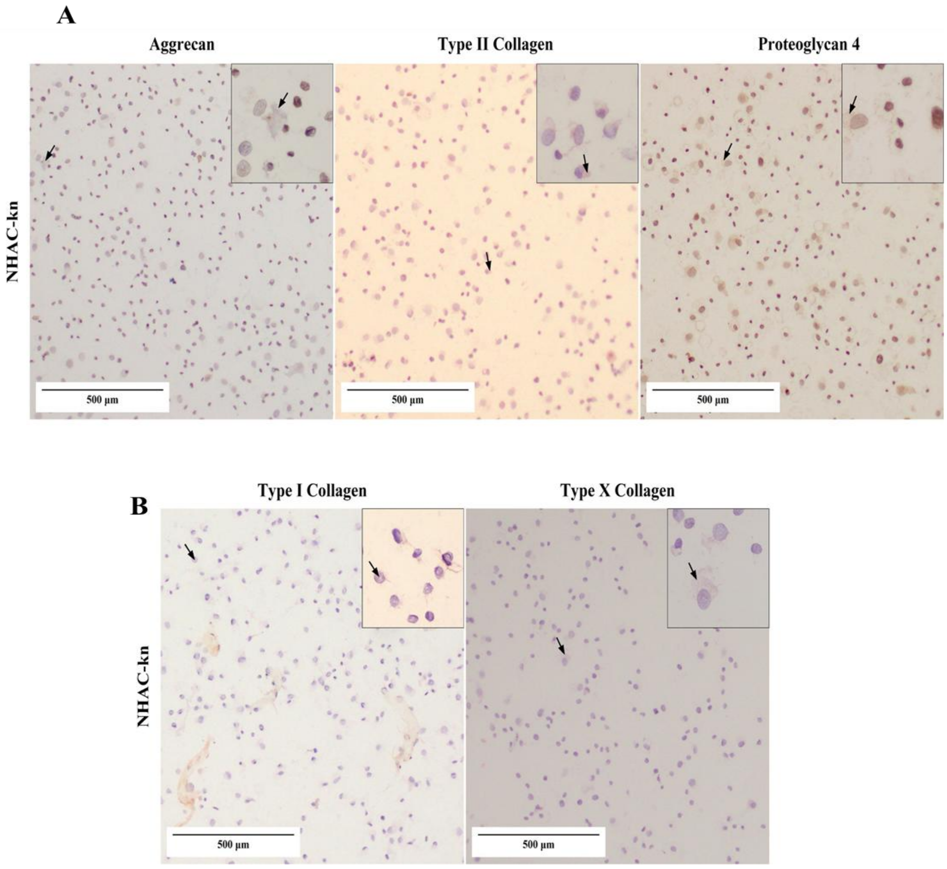


**Figure S2.** ECM Proteins in the NHAC-kn Cell Line. (A) Positive expression of aggrecan, type II collagen, and proteoglycan-4 in the NHAC-kn cell line. (B) Positive expression of type I collagen (+1) and negative expression of type X collagen in the NHAC-kn cell line. Images acquired using OLYMPUS DP22 microscope, 10X objective; inset at 40X objective.
